# Integrating “Super Skills for Exams” Programme in the School Curriculum to Support Adolescents Preparing for Their National Examinations in Turkey

**DOI:** 10.3390/children11020180

**Published:** 2024-02-01

**Authors:** Bilge Uzun, Asli Orman, Cecilia A. Essau

**Affiliations:** 1 Faculty of Educational Sciences, Bahcesehir University, 34353 Istanbul, Turkey; raziyebilge.uzun@bau.edu.tr; 2 Research Department, Ugur College, 34354 Istanbul, Turkey; asli.orman@ugurokullari.k12.tr; 3 School of Psychology, University of Roehampton, London SW15 4JD, UK

**Keywords:** examination stress, test anxiety, anxiety and depression, healthy lifestyles

## Abstract

Examination stress is the most common stressor reported by adolescents across the globe. Exam stress involves mental distress related to anticipated academic challenges or fear of failure in the examinations, test anxiety, or fear of being unable to meet certain expectations of themselves or others. The aim of this study was to determine the effectiveness of a transdiagnostic resilience program (Super Skills for Exams; SSE), when integrated in the school curriculum and delivered by the school counsellors, in reducing exam stress among adolescents who are preparing for their national examinations. SSE is based on the principles of Cognitive Behaviour Therapy (CBT), behavioural activation, and social skills training, and includes sessions in promoting healthy lifestyles. Participants were 7129 (3661 female and 3468 male) adolescents in grades 7 and 8, from all the 245 secondary schools from Ugur College in seven regions and 31 cities across Turkey. All the participants completed a set of questionnaires to measure self-efficacy for learning and test anxiety, academic stress, emotion regulation, and lifestyles. Results revealed significant reduction in academic stress (i.e., test anxiety, work pressure, self-expectation, and despondency) and maladaptive emotion regulation strategies, and significant increase in self-efficacy and adaptive regulation strategies following the intervention. Adolescents who participated in SSE reported an increase in the consumption of healthy food. This study provides preliminary empirical support for the integration of SSE within the school curriculum for helping adolescents cope with exam stress as they prepare for their national exams.

## 1. Introduction

Adolescence is a developmental stage that is associated with rapid physical growth and development in emotional, social, and cognitive abilities [1]. Adolescence is also associated with various important changes such as puberty, which can influence an adolescent’s self-image, personal identity, self-esteem, and confidence level. At the same time, adolescence is a sensitive period during brain development that is particularly susceptible to environmental input that contributes to adolescents’ increased risk for mental health problems, such as anxiety and depression [2]. As reported in numerous studies, the onset of most mental disorders is during adolescence [3], with adolescents who have been exposed to stress being at more elevated risk for developing mental health problems [4].

Stress occurs when an individual perceives that the demands of a situation exceed the individual’s personal, psychological, or social resources. As defined by Lazarus and Folkman [5] (1984, p. 19) “psychological stress is a particular relationship between the person and the environment that is appraised by the person as taxing or exceeding his or her resources and endangering his or her well-being”. Thus, the extent to which stress could have a negative impact on individuals’ functioning depends on the way the threat is perceived and the perception that they have inadequate resources to cope with the situations or adversities [5].

Of all the stressors, examination stress is the most frequently reported by adolescents [6]. Adolescents across the globe take exams at various times throughout their adolescence, which they may find very stressful and difficult [7]. Exam stress involves mental distress related to anticipated academic challenges or fear of failure in the examinations, test anxiety [7], or fear of being unable to meet certain expectations of themselves or others, and as such it can negatively affect across various areas of adolescents’ lives [8]. Exam stress not only has a negative impact on adolescent academic performance, but it could also lead to physical and mental health problems, suicidal intention, school absenteeism or dropout, and psychosocial impairment later in life (e.g., [9,10]). Other studies have shown exam stress to be associated with maladaptive behavioural coping responses such as smoking and alcohol intake [11].

Factors that are commonly related to exam stress include parents’ expectations, lack of physical and extracurricular activities, and test anxiety [12,13]. Those with test anxiety tend to have problems in preparing for exams, such as an inability to concentrate and revise study material effectively, and problems during the exam itself, such as an inability to focus key content and “going blank”; these problems often lead to exam stress, exam failures, and eventually school dropout [14]. Compared to male students, females were reported to experience higher levels of worry and test anxiety than their male counterparts [15]. Changing lifestyle habits and problem-solving training have been recommended to help reduce test anxiety [16].

Although exam stress is a common problem globally and across cultures, this type of stress is particularly high in Turkey where education is highly valued and thus the expectations of parents, teachers, and adolescents themselves to excel academically can be a source of intense stress. Furthermore, Turkish people are expected to pursue academic success to achieve respect and social mobility, resulting in extremely high pressure on children and adolescents.

The Turkish school system differs from other Organisation for Economic Co-operation and Development (OECD) countries in at least two ways. First, students choose their upper secondary school in grade 8 at the age 13, which is earlier than most OECD countries. Second, student high school selection is based on a centralised placement system called the Transition from Elementary Schools to Secondary Schools Exam (“Temel Eğitimden Ortaöğretime Geçiş Sistemi”; TEOG). Under this system, students are placed in one of their uppersecondary school preferences based on their results from a centralised examination (70% of the final placement score) and their average score in lower secondary school classroom assessments (30%). Placement exams are very competitive, which create a high level of stress among students. Furthermore, the pressure of high-stakes exams has been reported as the main reason for disengagement and early dropout of students [17].

Despite the common occurrence of exam stress and its negative impact, psychosocial programs that teach adolescents skills to overcome exam stress are lacking. The ability to cope with exam stress requires a holistic approach by targeting cognitive and behavioural efforts to control or reduce stressful experiences [18] and in promoting a healthy lifestyle [19]. One way to counter the negative effects of exam stress is to offer a preventative skills-training program in schools that provides adolescents with skills and specific techniques tools to effectively cope with exam stress [18]. Such a program would help to prevent school dropout, improve academic performance, and prevent the development of mental disorders.

Thus, the overall aim of this study is to describe the co-adaption and implementation of the Super Skill for Exam (SSE) program and present some preliminary findings using SSE. The main research questions that are addressed are as follows:

(1) Are there any significant gender differences on test anxiety, educational stress, cognitive emotion regulation, and lifestyle?
(2) What are the effects of SSE in reducing test anxiety and academic stress?
(3) What are the effects of SSE in improving self-efficacy for learning and cognitive emotion regulation and healthy lifestyles?

It is hypothesised that there are gender differences on test anxiety, educational stress, cognitive emotion regulation and lifestyles. Specifically, girls compared to boys are expected to report higher levels of test anxiety and educational stress; girls are expected to have higher scores on maladaptive cognitive emotion regulation and lead healthier lifestyles than boys.

At post-intervention, the SSE programme would demonstrate improvements in reducing test anxiety and academic stress, and in enhancing self-efficacy for learning and cognitive emotion regulation and healthy lifestyle.

## 2. Method

### 2.1. Participants

A total of 7129 school children completed the pre- and post-test questionnaires. Of these participants, 3661 were female (51.4%) and 3468 participants were male (48.6%). The mean age was 12.6 years. Moreover, 2771 (38.0%) were in the 7th grade and 4418 (61.97%) were in the 8th grade. All the participants came from middle socioeconomic backgrounds and live with both parents.

### 2.2. Procedure

The participants were recruited from all the 245 secondary schools from Ugur College in seven regions and 31 cities across Turkey. Ugur College is a private co-educational school that offers programs for children and adolescents aged 13–19. The curriculum model at Ugur College schools is regulated by the Ministry of Education, with classes held from September to June 2022.

The study was approved by Bahçeşehir University’s psychology ethics committee. A letter describing the present study was sent to the parents and they were given the opportunity to inform the teachers if they wished their child to be excluded from the study. A Before conducting this study, question-and-answer session was organised for parents. Due to the large number of parents who wish to know more about SSE and the present study, the question-and-answer session was conducted live on YouTubeby the first and last authors of this article. The session was attended by 9700 parents.

Children’s participation in this research was voluntary and they could withdraw from the research at any time. The children were informed that their data would be kept confidential.

### 2.3. Super Skills for Exams

Super Skills for Exams (SSE) was co-adapted from the Super Skills for Life (SSL) program. SSL is a psychosocial program that was developed to prevent anxiety and depression in children and adolescents. SSL is based on several core principles. First, it targets common core risk factors of anxiety and depression, including low self-esteem, lack of social skills, and cognitive dysfunction [20,21,22]. Second, by using the principles of CBT, it helps children develop skills to cope with stress-provoking situations [23]. Third, it uses video feedback with cognitive preparation to help children enhance their self-perception and appraisal of their performance [24,25]. Fourth, by having children increase their activity levels and participate in positive activities, it will in turn help to improve the children’s mood and self-esteem. Finally, it teaches children skills to use during social interactions and techniques to solve social problems.

**Figure 1:**
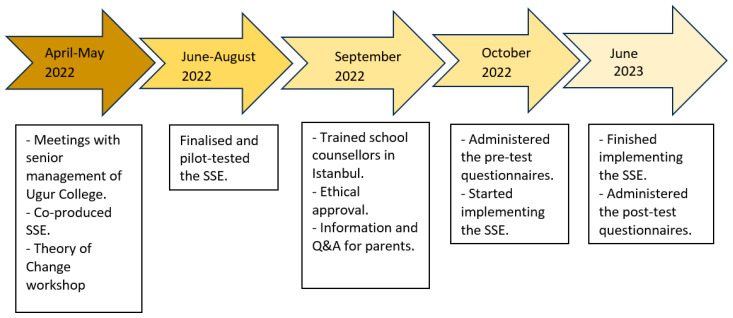
From co-design to implementation of Super Skills for Exams.

In May 2022, the senior author of SSL (C.A.E.) and the first author of this article (B.U.) were approached by Ugur College’s senior management to co-adapt SSL to provide skills and techniques that could help their students cope with exam stress (Figure 1). Ugur College wanted SSE to be delivered to all the adolescents who are preparing for their national examinations; this applies to all students in grades 7, 8, 10, and 11. For the present study, only data from adolescents in grades 7 and 8 were used. Ugur College also wanted SSE to be delivered throughout the academic year, which meant that SSL had to be expanded from 8 to 16 sessions (see Table 1). Throughout the co-adaption period, we received significant input from the teachers and senior management of Ugur College in terms of the contents of SSE, but also in terms of logistics in delivering SSE to Ugur schools.

### 2.4. Implementation of SSE

SSE was delivered by the school counsellors at each participating school after receiving an intensive two-day workshop in Istanbul by the two authors of this manuscript (C.A.E. and B.U.). Two hundred and seventy school counsellors participated in the workshop. The main aim of this workshop was to ensure that the school counsellors follow the SSE implementation protocol and to overcome potential problems in its implementation. The workshop covered topics related to exam stress, anxiety, and depression and their risk factors, and the principles of prevention. All the school counsellors were given a leader’s manual that included a detailed outline of each session of the SSE programme. Regular supervision (on average every two weeks) was given to all the school counsellors by the first author (B.U.).

As SSE is part of the Ugur College curriculum, it was delivered to all the adolescents in the classroom setting. Each session, which is about 45 min long, was delivered once a week. The adolescents received a copy of the SSE workbook. At the end of each session, the adolescents were given some home activities that involved practicing the skills they had learnt in the session.

### 2.5. Measures

The adolescents completed a set of questionnaires before and after the intervention.

The Motivated Strategies for Learning Questionnaire (MSLQ) [26] was used to assess self-efficacy for learning and test anxiety. MSQL originally included 56 items that are rated on a 7-point Likert scale (1 = not all true for me, 7 = very true for me). It has 6 subscales, namely intrinsic value, extrinsic value, self-efficacy, test anxiety, strategy use, and self-regulation. To reduce the number of items in the entire data collection tool, only the most relevant subscales (test anxiety and self-efficacy) for the present study were included. In the original study, internal consistency of the scale and subscales showed good reliability; the Cronbach alpha for self-efficacy was 0.89, and for test anxiety subscale, it was 0.75. In the present study, the Cronbach alpha was 0.84 for test anxiety and 0.62 for the self-efficacy subscales, which indicated good and acceptable reliability, respectively.

The Education Stress Scale for Adolescents (ESSA) [27] was used to measure academic stress. The EESA has 16 items that can be rated on a 5-point Likert scale (1 = never true to 5 = almost always true). Its items were divided into five subscales, namely, pressure from study, workload, worry about grades, self-expectation, and despondency. The original study showed good reliability scores in that the Cronbach alpha for the total scale was 0.81. For the five subscales, the Cronbach alpha ranged from 0.62 (despondency) to 0.75 (pressure from study). The Turkish version of the scale produced four factors [28]. Similar to the original study, reliability analyses for the current sample revealed good Cronbach alphas (α = 0.85) for the total scale and for the pressure from study, workload, self-expectation, and despondency subscales (57, 0.62, 0.77, and 0.59, respectively).

The Cognitive Emotional Regulation Questionnaire (CERQ) [29] was used to measure cognitive aspects of emotion regulation. Each of its 18 items can be rated on a 5-point Likert scale, ranging from 1 = almost never to 5 = almost always. The items were grouped into two subscales (maladaptive coping strategies and adaptive coping strategies). The internal reliability of CERQ was 0.83 [30] for the total scores; the internal consistency in the present study was 0.75 for the total score.

The Adolescent Lifestyle Profile—Revised 2 (ALP R2) [31] was used to determine health-promoting behaviours. ALP R2 consists of 44 items which can be rated on a 4-point scale (1 = never, 4 = always); it has seven subscales, namely, health responsibility, physical activity, nutrition, positive life perspective, interpersonal relationship, stress management, and spiritual health. The interpersonal relationship and spiritual health subscales were considered not directly relevant to the present study; therefore, these two subscales were removed. As in the original study, the Cronbach alpha for the total scores was good (α = 0.79).

Sociodemographic Questionnaire included the questions regarding adolescents’ gender, age, grade, and living status (with or without parents).

### 2.6. Statistical Analysis

Preliminary analyses were conducted to determine the univariate and multivariate normality of the sample. The skewness and kurtosis coefficients were used to examine the distribution of study variables for both pre- and post-tests. The coefficients calculated for the scores obtained from the questionnaires (−0.85.8 ≤ Skewness ≤ 0.95, −0.89 ≤ Kurtosis ≤ 1.58) were within the specified range. Data were analysed using SPSS 25.0. The analyses were performed on each variable by including their subscales in order to control Type 1 error. The study was conducted during two semesters in the 2022–2023 academic year. Since the data were collected from a total of 245 schools across Turkey, the long time between pre- and post-test data, and the large size of the sample caused the data to become independent; an independent sample *t*-test was used in the analysis of this study.

## 3. Results

### 3.1. Pre-Treatment Comparisons

A series of two-way (gender × grade) MANOVA’s was carried out on pre-test data to determine if there were any significant differences in participants’ scores on the following variables: test anxiety and self-efficacy; pressure from study, workload, self-expectation, and despondency; maladaptive and adapting coping strategies; and lifestyles, including physical activity, nutrition, stress management, and health responsibility (obtained from ASL). Results of the descriptive statistics are presented in Table 1.

On Motivational Strategies and Learning, two-way MANOVA (gender × grade) results showed no interaction effect (λ = 0.998; *F*_2.3562_ = 2.96; *p* > 0.05, partial η^2^ = 0.002). Univariate analysis showed a significant main effect of gender difference on test anxiety (*F*_1.3566_ = 179.49; *p* < 0.05, partial η^2^ = 0.05) and self-efficacy (*F*_1.3566_ = 69.01; *p* < 0.05, partial η^2^ = 0.02), indicating that females (M = 16.56; SD = 6.41) reported higher test anxiety than males (M = 13.58; SD = 6.09). Similarly, they had (M = 36.23; SD = 6.74) higher self-efficacy than males (M = 34.19; SD = 7.61).

On Educational Stress, no significant gender–grade interaction effect was found (λ = 0.997; *F*_4.3560_ = 2.35; *p* > 0.05, partial η^2^ = 0.003). However, univariate analysis showed a main effect of gender on work pressure (*F*_1.3566_ = 16.92; *p* < 0.05, partial η^2^ = 0.005), worry about grades (*F*_1.3566_ = 6.89; *p* < 0.05, partial η^2^ = 0.002), self-expectations (*F*_1.3566_ = 150.48; *p* < 0.05, partial η^2^ = 0.041), and despondency (*F*_1.3566_ = 102.07; *p* < 0.05, partial η^2^ = 0.028). Specifically, females reported higher work pressure (M = 13.60; SD = 3.42), worry about grades (M = 9.62; SD = 3.21), self-expectations (M = 19.46; SD = 4.32), and despondency (M = 12.28; SD = 3.71) then males.

Similarly, univariate analysis showed a main effect of grade on work pressure (*F*_1.3566_ = 12.45; *p* < 0.05, partial η^2^ = 0.003), worry about grades (*F*_1.3566_ = 9.27; *p* < 0.05, partial η^2^ = 0.003), self-expectations (*F*_1.3566_ = 3.86; *p* < 0.05, partial η^2^ = 0.001), and despondency (*F*_1.3566_ = 8.22; *p* < 0.05, partial η^2^ = 0.001). These findings showed that 8th graders reported higher work pressure (M = 13.51; SD = 3.42), worry about grades (M = 9.61; SD = 3.23), self-expectations (M = 18.59; SD = 4.58), and despondency (M = 11.76; SD = 3.82) when compared to 7th graders.

On Cognitive Emotion Regulation, results of the two-way (gender × grade) MANOVA showed significant interaction effect (λ = 0.998; *F*_2.3562_ = 3.94 *p* < 0.05, partial η^2^ = 0.002). Univariate analysis showed gender–grade interaction on adaptive coping strategies (*F*_1.3566_ = 5.83; *p* < 0.05, partial η^2^ = 0.002). These findings suggested that 8th-grade males showed the highest score (M = 33.84; SD = 6.43) on adaptive coping strategies when compared to the other groups (8th-grade females and 7th-grade males and females).

On Adolescent Lifestyle, results showed significant interaction effect of gender × grade (λ = 0.990; *F*_4.3560_ = 9.22 *p* < 0.05, partial η^2^ = 0.010). Univariate analysis showed gender–grade interaction only on nutrition (*F*_1.3566_ = 7.26; *p* < 0.05, partial η^2^ = 0.002) and health responsibility (*F*_1.3566_ = 8.92; *p* < 0.05, partial η^2^ = 0.002). Specifically, 7th-grade males reported highest (M = 20.11; SD = 3.47) nutrition and health responsibility (M = 16.53; SD = 4.46) when compared to the other groups (7th-grade males and 8th-grade female and males).

### 3.2. Intervention Effect

In terms of Motivational Strategies and Learning, results of the independent sample *t*-test showed a significant effect of SSE on total group scores on test anxiety (*t* = 5.58, *p* < 0.005) and self-efficacy (*t* = 11.25, *p* < 0.005). This finding suggested that SSE was effective in reducing test anxiety and in increasing self-efficacy. Specifically, results revealed that male’s test anxiety post-test scores (M = 12.89, SD = 6.19) were lower than their pre-test scores (M = 13.58, SD = 6.09); whilst, their self-efficacy post-test (M = 34.19, SD = 7.61) scores were higher than their pre-test scores (M = 31.73, SD = 8.04). In a similar vein, females’ post-test scores (M = 15.47, SD = 6.63) were significantly lower than their pre-test scores (M = 16.56, SD = 6.41) on test anxiety, whilst their post-test scores were higher (M = 36.23, SD = 6.74) than their pre-test scores (M = 34.61, SD = 7.11) on self-efficacy. Results are presented in Table 2.

On Educational Stress, SSE was effective in reducing work pressure (*t* = 12.59, *p* < 0.005), self-expectation (*t* = 7.20, *p* < 0.005), and despondency (*t* = 3.65, *p* < 0.005). No significant effect of the program on worry about grades (*t* = 1.31, *p* > 0.005) was found.

On Cognitive Emotion Regulation, results of the independent sample *t*-test on pre- and post-test scores showed the SSE programme was effective in increasing adaptive regulation strategies (*t* = 7.20, *p* < 0.005) and in reducing maladaptive regulation strategies (*t* = 3.22, *p* < 0.005).

After participating in the SSE program, the children consumed more healthy food (*t* = 4.27, *p* < 0.005) and were better able to manage their stress (*t* = 2.11, *p* < 0.005). However, no significant increase in physical activity (*t* = 1.89, *p* > 0.005) or health responsibility (*t* = 1.21, *p* > 0.005) was found.

### 3.3. Intervention Effect by Gender

To examine the effect of SSE on the study variables, a series of independent sample *t*-tests were performed on males and females (Table 2).

Motivational Strategies and Learning: SSE was effective in reducing test anxiety (*t* = 3.29, *p* < 0.005) and in increasing self-efficacy (*t* = 9.23, *p* < 0.005) in males as well as in females (test anxiety (*t* = 5.05, *p* < 0.005); self-efficacy (*t* = 7.05, *p* < 0.005)).

On Educational Stress, SSE was found effective on the following subscales: work pressure (*t* = 9.01, *p* < 0.005), self-expectation (*t* = 5.97, *p* < 0.005), and despondency (*t* = 3.44, *p* < 0.005) among males. Among females, the results also showed a significant reduction in work pressure (*t* = 8.96, *p* < 0.005) and self-expectation (*t* = 4.78, *p* < 0.005) subscales. SSE did not result in a significant reduction in worry about grades among males (*t* = 0.83, *p* > 0.005) and females (*t* = 1.08, *p* > 0.005).

SSE was effective in increasing adaptive regulation strategies among males (*t* = 6.09, *p* < 0.005) and females (*t* = 4.05, *p* < 0.005). However, no significant reduction was found in maladaptive regulation strategies among males (*t* = 2.63, *p* > 0.005) and females (*t* = 4.05, *p* < 0.005).

SSE was found effective in improving consumption of healthy food (*t* = 3.83, *p* < 0.005) and in stress management (*t* = 3.21, *p* < 0.005) among males. However, this significant finding was not replicated among females (nutrition (*t* = 2.11, *p* > 0.005); stress management (*t* = 0.14, *p* > 0.005)). Among males, there were no significant effects of SSE on physical activity (*t* = 1.63, *p* > 0.005) and health responsibility (*t* = 2.39, *p* > 0.005). Among females, no significant effects of SSE were found on, physical activity (*t* = 0.87, *p* > 0.005), health responsibility (*t* = 0.73, *p* > 0.005).

## 4. Discussion

The objective of this study was to examine the impact of the Super Skills for Exam (SSE) program in reducing exam stress and test anxiety among adolescents who were preparing for their national examination in Turkey. For this purpose, the Super Skills for Life programme [32] was co-adapted and integrated in the school curriculum for 7th and 8th graders in Turkey’s Ugur College. The co-adapted program (called Super Skills for Exams) was expanded from 8 to 16 weeks and implemented over two semesters (fall and spring) during the 2022–2023 academic year. A total of 7129 adolescents completed a set of questionnaires one week before and one week after the intervention.

The findings can be summarised as follows: First, in line with previous studies, females had higher test anxiety, worry about their grades, self-expectations, and despondency than males [33,34,35,36]. The reason for this gender difference is unclear, although differences in gender socialisation have been proposed [36]. In this context, it has been argued that girls are allowed to express their fears and worries and that they are more frequently oriented towards dependence, fearfulness, passivity, and obedience compared to boys [33,37]. In contrast, boys are more likely to be taught to contain feelings of fear and insecurity; boys are also socialised to develop attitudes and behaviours that are associated with a masculine role related to problem-solving and goal achievement [38]. Cultural issues may also account for these gender differences. Turkey is influenced greatly by both Eastern and Western culture characterised by collectivistic society [39]. Turkish parents let their sons behave more independently and aggressively whereas daughters are expected to be more dependent and obedient [40]. These differences are manifested through the work roles for adolescents in the academic setting for the students [41]. Considering this assumption, the differences between girls’ and boys’ test anxiety, worry about their grades, self-expectations, and despondency seem reasonable.

Studies that have examined the role of puberty on negative emotions such as sadness have reported no gender differences. For example, in a study by Susman and colleagues [42], girls 9 to 14 years old with earlier maturation (based on gonadotropin hormone levels) reported higher sadness scores compared to same-age girls with later maturation. Among boys with earlier compared to later maturation (based on adrenal androgen levels), higher sadness scores were similarly reported.

Second, adolescents in grade 8 reported higher academic stress (i.e., work pressure, worry about their grades, self-expectations, and despondency) compared to those in grade 7. This finding is not surprising because the 8th graders are going to do the exam at the end of the semester, while as those in grade 7 have one more year to prepare for their national exam. While more future studies are needed, our finding on the high levels of academic stress among 8th-grade students suggests the need to consider abolishing national examinations to be taken by students at a young age. In terms of lifestyle, males in grade 7 reported the highest scores on nutrition and health responsibility. Previous studies showed mixed findings on gender differences in nutrition and health responsibility. For example, among adolescents in Indonesia, males compared to females were more likely to choose healthy foods [43]. Other studies have found female adolescents to be more concerned with their dietary behaviours; this concern is related to female tendency to give more attention to their body image or body shape [44]. In line with Albani Zambon et al.’s study [45], we found males in grade 7 to have the highest scores on nutrition. Furthermore, a study by Maulida and colleagues [43] found that fruit intake began to decrease at the age of 7 years and reached its lowest level during adolescence.

Third, in line with previous studies, e.g., [46,47], participation in SSE led to a significant reduction in maladaptive emotion regulation strategies, which were significantly reduced at post-intervention assessment. SSE also led to a significant reduction in academic stress (i.e., test anxiety, work pressure, self-expectation, and despondency) and significant increases in self-efficacy and adaptive regulation strategies. In SSE, adolescents are taught skills to challenge worry cognitions (e.g., negative beliefs about their academic abilities) and avoidance behaviours.

Fourth, adolescents who participated in SSE reported an increase in the consumption of healthy food. This is an important finding given the positive impact of healthy food on the mental health of adolescents. As shown in numerous studies, diet quality was associated with adolescent mental health, and that improvements in healthy diet were associated with improvements in mental health, while reductions in healthy diet were linked with a decline in psychosocial impairment over the follow-up period, e.g., [48]. To our knowledge, programs that have been developed to overcome academic/exam stress among adolescents rarely include healthy lifestyles. In SSE, in addition to teaching adolescents the cognitive–behavioural skills to overcome exam stress, it also targets lifestyles to ensure that adolescents are physically well by eating healthy food, remaining physically active, and achieving sleep hygiene.

In interpreting our findings, it is important to consider the study’s limitations. First, all the participants were students attending Ugur College, which is a chain school in 254 cities across Turkey, and from middle socioeconomic backgrounds. Thus, the sample would not be representative to all other adolescents. Furthermore, the present study used an open trial design; thus, the internal validity of SSE could potentially be explained by the passage of time that may not be related to the program. Future studies should therefore consider conducting a randomised control trial to determine if the present findings can be replicated. Furthermore, SSE was delivered by the school counsellors in each participating school. No therapist treatment integrity measurements were included; thus, it is not clear the extent to which the facilitators strictly followed the intervention protocol. Another limitation might be related to the statistical analysis. Although the amount of the pre- and post-test data was huge, they were uncontrollable and lowered the effect size. Thus, a methodological weakness was the lack of statistical power, which in part may be due to uncontrolled variables or confounding factors.

Notwithstanding these caveats, adolescents who participated in the SSE programme showed a reduction in academic stress and maladaptive emotion regulation strategies.

## 5. Conclusions

To our knowledge, this is the first study to have integrated a CBT-based psychological intervention in a school curriculum and delivered by the school counsellors of the participating schools. Our findings, parallel to our aim, have important implication for educational and public health policies in relation to supporting adolescents during their national exam year. Specifically, the SSE program embedded in the academic curriculum had effect on decreasing test anxiety and increasing self-esteem.

## Figures and Tables

**Table 1:** Descriptive statistics of the study variables.

		7th Grade				8th Grade			
	Variables	Male N = 668		Female N = 722		Male N = 1070		Female N = 1087	
		M	(SD)	M	(SD)	M	(SD)	M	(SD)
Motivational Strategies and Learning	Test Anxiety	14.07	(6.02)	16.43	(6.35)	13.27	(6.12)	16.65	(6.46)
	Self-Efficacy	34.18	(7.71)	36.25	(6.86)	34.19	(7.55)	36.22	(6.66)
Educational Stress	Study Pressure	12.94	(3.43)	13.25	(3.41)	13.19	(3.41)	13.84	(3.40)
	Workload	9.13	(3.25)	9.39	(3.12)	9.45	(3.19)	9.76	(3.26)
	Self-expectation	17.52	(4.92)	19.04	(4.46)	17.44	(4.65)	19.74	(4.21)
	Despondency	10.90	(3.67)	11.89	(3.49)	10.97	(3.64)	12.54	(3.83)
Cognitive Emotion Regulation	Maladaptive Coping Strategies	23.44	(6.45)	24.76	(6.34)	24.17	(6.18)	25.86	(6.10)
	Adaptive Coping Strategies	33.49	(6.64)	33.72	(6.52)	33.84	(6.43)	33.02	(6.08)
Lifestyle	Physical Activity	17.39	(4.15)	16.38	(4.33)	16.27	(4.53)	14.83	(4.50)
	Nutrition	20.11	(3.31)	19.25	(3.47)	19.84	(3.25)	18.36	(3.60)
	Health Responsibility	16.53	(4.46)	15.63	(4.17)	15.49	(4.30)	15.46	(4.15)
	Stress Management	18.37	(3.28)	18.32	(3.26)	17.80	(3.36)	18.12	(3.36)

**Table 2:** Study variables before and after participating in the Super Skills for Exam programme.

		All Sample				Male				Female			
	Variables	Pre-Test N = 3567		Post-Test N = 3562		Pre-Test N = 1758		Post-Test N = 1710		Pre-Test N = 1809		Post-Test N = 1852	
		M	(SD)	M	(SD)	M	(SD)	M	(SD)	M	(SD)	M	(SD)
Motivational Strategies and Learning	Test Anxiety	15.09	(6.43)	14.23	(6.55)	13.58	(6.09)	12.89	(6.19)	16.56	(6.41)	15.47	(6.63)
	Self-Efficacy	33.23	(7.71)	35.22	(7.25)	31.73	(8.04)	34.19	(7.61)	34.61	(7.11)	36.23	(6.74)
Educational Stress	Study Pressure	13.36	(3.43)	12.31	(3.51)	13.09	(3.42)	12.01	(3.56)	13.60	(3.42)	12.59	(3.44)
	Worry about Grades	9.47	(3.22)	9.37	(3.20)	9.32	(3.16)	9.32	(3.16)	9.62	(3.21)	9.50	(3.24)
	Self-expectation	18.48	(4.65)	17.65	(4.99)	17.47	(4.75)	16.48	(4.97)	19.46	(4.32)	18.74	(4.75)
	Despondency	11.62	(3.74)	11.30	(3.81)	10.95	(3.65)	10.52	(3.64)	12.28	(3.71)	12.01	(3.83)
Cognitive Emotion Regulation	Adaptive Coping Strategies	32.38	(6.68)	33.50	(6.39)	32.32	(6.81)	33.70	(6.51)	32.44	(6.56)	33.30	(6.27)
	Maladaptive Coping Strategies	24.66	(6.30)	24.19	(6.21)	23.89	(6.29)	23.34	(6.22)	25.42	(6.22)	24.97	(6.09)
Lifestyle	Physical Activity	16.07	(4.50)	15.87	(4.58)	16.71	(4.42)	16.46	(4.48)	15.45	(4.49)	15.32	(4.61)
	Nutrition	18.96	(3.60)	19.32	(3.48)	19.50	(3.54)	19.94	(3.28)	18.71	(3.57)	18.46	(3.58)
	Health Responsibility	15.71	(4.41)	15.59	(4.41)	15.90	(4.39)	15.54	(4.49)	15.53	(4.16)	15.63	(4.34)
	Stress Management	18.12	(3.33)	17.95	(3.46	17.65	(3.56)	18.02	(3.34)	18.20	(3.32)	18.22	(3.34)

## Data Availability

The data presented in this study are available on request from the corresponding author. The data are not publicly available due to ethical reasons.
